# Genotoxic stress causes the accumulation of DNA‐dependent protein kinase catalytic subunit phosphorylated at serine 2056 at nuclear speckles and alters pre‐mRNA alternative splicing

**DOI:** 10.1002/2211-5463.12569

**Published:** 2018-12-28

**Authors:** Shuang Liu, Yuan Shao, Qi Wang, Yonggong Zhai, Xialu Li

**Affiliations:** ^1^ Beijing Key Laboratory of Gene Resource and Molecular Development College of Life Sciences Beijing Normal University China; ^2^ Beijing Key Laboratory of DNA Damage Response College of Life Sciences Capital Normal University China

**Keywords:** DNA damage response, DNA‐PKcs, genotoxic stress, nuclear speckles, pre‐mRNA splicing

## Abstract

RNA splicing has emerged as a critical player in the DNA damage response (DDR). However, the underlying mechanism(s) by which pre‐mRNA splicing is coordinately regulated by genotoxic stress has remained largely unclear. Here, we show that a DDR factor, DNA‐dependent protein kinase (DNA‐PK), participates in the modulation of pre‐mRNA splicing in the presence of DNA double‐strand break (DSB)‐induced genotoxic stress. Through indirect immunostaining, we made the surprising discovery that DNA‐PK catalytic subunits (DNA‐PKcs) autophosphorylated at serine 2056 (S2056) accumulate at nuclear speckles (dynamic nuclear structures that are enriched with splicing factors), following their dissociation from DSB lesions. Inactivation of DNA‐PKcs, either using a small molecule inhibitor or by RNA interference, alters alternative splicing of a set of pre‐mRNAs in A549 cells treated with the topoisomerase II inhibitor mitoxantrone, indicative of an involvement of DNA‐PKcs in modulating pre‐mRNA splicing following genotoxic stress. These findings indicate a novel physical and functional connection between the DNA damage response and pre‐mRNA splicing, and enhance our understanding of how mRNA splicing is involved in the cellular response to DSB lesions.

**Abbreviations**
53BP1: p53‐binding protein 1
AS: alternatively spliced
ATM: ataxia telangiectasia mutated
CPT: camptothecin
CS: constitutively splcied
DAPI: 4′,6‐diamidino‐2‐phenylindole
DDR: DNA damage response
DNA‐PKcs: DNA‐dependent protein kinase catalytic subunit
DNA‐PK: DNA‐dependent protein kinase
DSB: DNA double‐strand break
Etop: etoposide
GAPDH: glyceraldehyde 3‐phosphate dehydrogenase
γ‐H2AX: Histone H2AX phosphorylated at serine 139
hnRNP: heterogeneous nuclear ribonucleoprotein
MTX: mitoxantrone
NCS: neocarzinostatin
NHEJ: non‐homologous end joining
PTC: premature termination codon
shRNA: short hairpin RNA
Top II: topoisomerase II

Double‐strand breaks (DSBs) are considered the most detrimental type of DNA lesion. A single DSB, if unrepaired, can trigger cell growth arrest and cell death, whereas improperly repaired DSBs may induce gross chromosomal rearrangements and lead to malignant transformation via the activation of oncogenes and/or the loss of tumor suppressors 1. To protect genome integrity, all organisms have evolved complex responding systems to sense, signal and repair DSB lesions 2. In mammalian cells, DSBs are predominantly repaired by the non‐homologous end joining (NHEJ) pathway, which mediates the direct re‐ligation of the broken DNA ends independent of the presence of a homologous repair template 3. DNA‐dependent protein kinase (DNA‐PK), a member of the phosphoinositide 3‐kinase‐related kinase family, is indispensable for the initiation of the NHEJ‐mediated DSB repair pathway 4. DNA‐PK holoenzyme consists of the Ku70/80 heterodimer and a 470‐kDa catalytic subunit (DNA‐PKcs) with serine/threonine protein kinase activity. The activation of the DNA‐PK complex requires simultaneous binding of the holoenzyme to DSBs, which initiates the NHEJ process 4. In the current model of the NHEJ process, the recruitment of DNA‐PKcs to a damage site is essential for the establishment of a proper juxtaposition of broken ends 5. The DNA‐PK–DNA complex is subsequently destabilized by a set of phosphorylation events on the DNA‐PKcs, which significantly increases the accessibility of DNA ends to the XRCC4‐ligase IV complex in the NHEJ process 6, 7, 8.

DNA‐PKcs is phosphorylated on more than 40 sites in the presence of DSBs 4. Among these sites, phosphorylation on the threonine 2609 (T2609) cluster and the serine 2056 (S2056) cluster are best characterized and essential for DNA‐PK‐mediated DSB repair. The T2609 cluster is primarily phosphorylated by ataxia telangiectasia mutated (ATM) in response to DSBs 9, whereas S2056 is a bona fide DSB‐induced autophosphorylation site *in vivo* 10, 11. Although the phosphorylation on both clusters is dispensable for the catalytic activity of DNA‐PKcs and its recruitment at DSBs 11, phosphorylation facilitates the dissociation of DNA‐PKcs from DNA ends at damage sites, likely due to phosphorylation‐dependent conformational change(s), which alters the affinity of the DNA‐PKcs for the Ku70/80 heterodimer and DNA ends 6, 7, 8, 12.

Despite these advances in our understanding of how the phosphorylation status affects the functionality of DNA‐PKcs in DSB repair, relatively little is known about the fate of phosphorylated DNA‐PKcs following its dissociation from DNA damage sites. Here, we show that autophosphorylated DNA‐PKcs at S2056 is redistributed to nuclear speckles, highly likely occurring after it is excluded from DNA damage sites. Indicative of a potential biological significance for the translocation of DNA‐PKcs to speckles, DNA‐PK inactivation, by both a small molecule inhibitor and RNA interference, affects pre‐mRNA alternative splicing in A549 cells in the presence of the DSB‐inducer mitoxantrone (MTX). Taken together, these data indicate an unappreciated function of DNA‐PK, a principal DNA damage response (DDR) factor involved in the NHEJ pathway, in modulating pre‐mRNA splicing in response to genotoxic stress in human cells.

## Materials and methods

### Cell culture and lentivirus‐based transduction

A549 and U2OS cells (ATCC, Manassas, VA, USA) were maintained in RPMI 1640 medium supplemented with 10% fetal bovine serum (PAN Biotech, Aidenbach, Germany) and 2 mM GlutaMAX (Thermo Fisher Scientific, Waltham, MA, USA) at 37 °C with 5% CO_2_. Lentiviruses were prepared following the Lentiweb Protocol (https://www.addgene.org/protocols/lentivirus-production/) and were used directly to transduce cells in the presence of 8 μg·mL^−1^ polybrene. Cells were analyzed 48 h post‐transduction.

### Indirect immunofluorescence staining

A549 or U2OS cells were fixed with 4% paraformaldehyde for 10 min at room temperature. The cells were then permeabilized with 0.5% Triton X‐100 in PBS for 10 min and blocked with 5% bovine serum albumin in PBS for 30 min. Primary antibodies were incubated for 1 h at room temperature. Secondary antibodies (Sigma‐Aldrich, St. Louis, MO, USA) were incubated for 45 min at room temperature. The cells were subsequently counterstained with 4′,6‐diamidino‐2‐phenylindole (DAPI) for 10 min to stain the nucleus. Z‐stack and max intensity projection images were generated with a Nikon ECLIPSE Ni microscope (Nikon, Minato, Tokyo, Japan) and nis‐element software (advanced research version, Nikon).

The primary antibodies used were as follows: anti‐DNA‐PKcs phospho S2056 (ab18192; Abcam, Cambridge, UK), anti‐Histone H2AX phospho S139 (γ‐H2AX, 05636; Millipore, Burlington, MA, USA), anti‐p53‐binding protein 1 (53BP1; MAB3802; Millipore; sc‐22760; Santa Cruz Biotechnology, Dallas, TX, USA), anti‐ATM phospho‐S1981 (4526S; Cell Signaling Technology, Danvers, MA, USA), and anti‐SRSF2 (ab11826; Abcam).

### RNA and protein analyses

Total RNA purification, reverse transcription PCR (RT‐PCR) and western blot were performed as described 13. The image of the agarose gel electrophoresis was quantified by Quantity One (Bio Rad, Hercules, CA, USA). Real‐time quantitative RT‐PCR (qRT‐PCR) was performed using SYBR Premix Ex Taq (TaKaRa, Shiga, Japan) in the CFX‐96 Real‐time fluorescence PCR detection system (Bio‐Rad, Hercules, CA, USA).

The primary antibodies for western blot were as follows: anti‐DNA‐PKcs (sc‐9051; Santa Cruz Biotechnology), anti‐DNA‐PKcs phospho S2056 (ab18192; Abcam), and anti‐glyceraldehyde 3‐phosphate dehydrogenase (GAPDH; 60004‐1‐Ig; Proteintech, Rosemont, IL, USA).

The primers used for PCR were as follows: *SRSF1*‐Exon1‐FP (5′‐CTTTTCGTCACCGCCA TGTC‐3′) and *SRSF1*‐Exon1‐RP (5′‐TTGGTTCGGATGTCTGGAGG‐3′); *SRSF1*‐alternatively spliced (AS) ‐FP (5′‐GG AGGCXAATGGTTTGGATTGG‐3′) and *SRSF1*‐AS‐RP (5′‐CCACACGAATGCGGTTTGG‐3′); *SRSF1*‐constitutively spliced (CS)‐FP (5′‐GTTGTCTCTGGACTGCCTCC‐3′) and *SRSF1*‐CS‐RP (5′‐GACACCAGTGCCATCTCGG‐3′); *SRSF1*‐FP (5′‐CCTGGTTTGTGTGAACTGGG‐3′) and *SRSF1*‐RP (5′‐ CCAATGATCCAGCTCCAAGG‐3′); *SRSF2*‐FP (5′‐TCCAAGTCCAAGTCCTCGTC‐3′) and *SRSF2*‐RP (5′‐TCCCCAAGTCCTCCGTTTAC‐3′); *HNRNPDL*‐FP (5′‐CAGAGCACTTATGG‐CAAGGC‐3′) and *HNRNPDL*‐RP (5′‐TGACGCAGAAAAGCAATCACA‐3′); *HNRNPH1*‐FP (5′‐AATAGTCCTGACACGGCCAA‐3′) and *HNRNPH1*‐RP (5′‐CAGCCCCAGGTCTGTCATAA‐3′); *PRPF38B*‐FP (5′‐ACCTCAACCCCATGATCCTG‐3′) and *PRPF38B*‐RP (5′‐TGCTTCCTTTC‐TCCCATGGT‐3′).

## Results

### S2056‐phosphorylated DNA‐PKcs accumulates at discrete nuclear foci outside of DNA damage sites in MTX‐treated A549 cells

**Figure 1:**
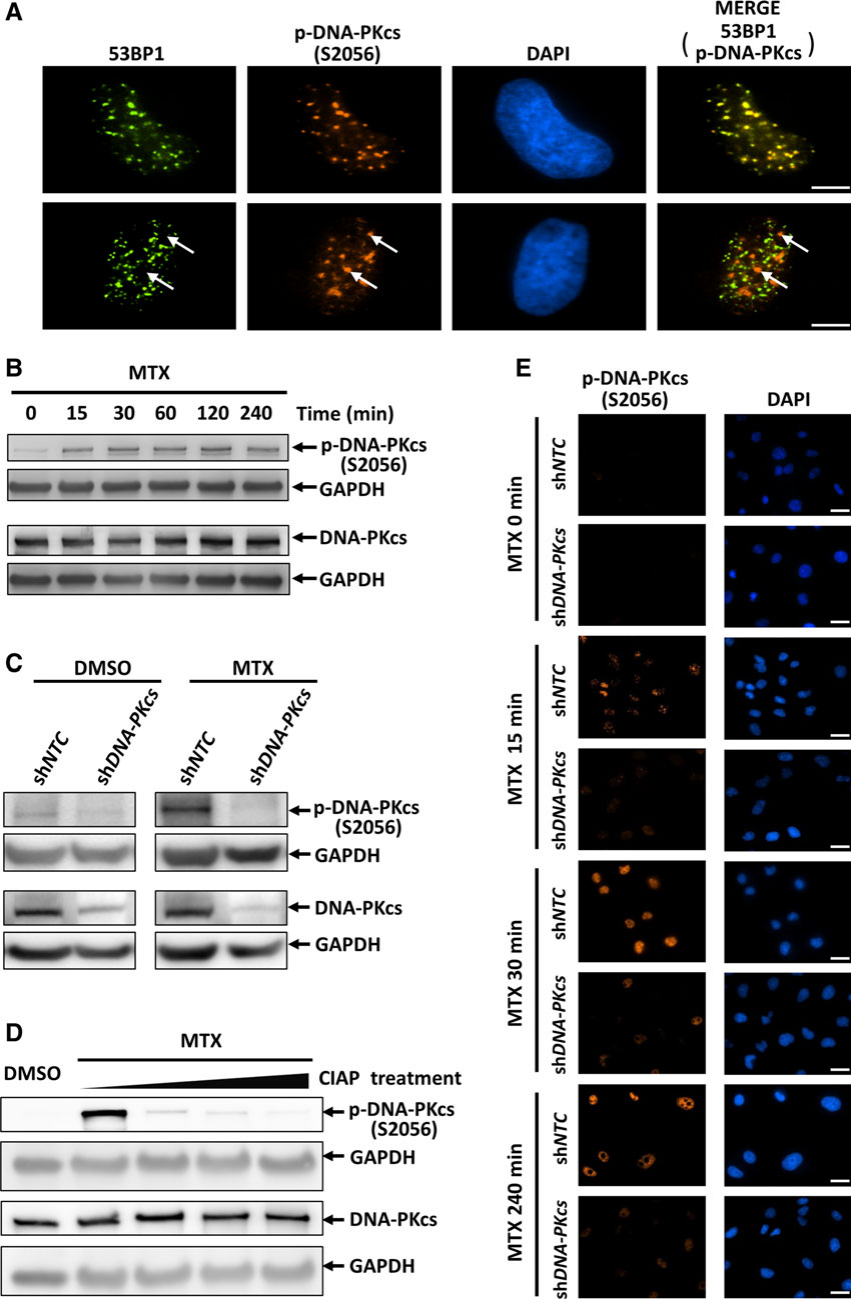
S2056‐phosphorylated DNA‐PKcs accumulates at discrete nuclear foci outside of DNA damage sites in MTX‐treated A549 cells. (A) A549 cells were treated with 10 μm MTX for 60 min and subjected to indirect immunofluorescence staining for phosphorylated DNA‐PKcs (p‐DNA‐PKcs) (S2056) and 53BP1 as indicated. The images in the upper panel are representative of cells in which S2056‐phosphorylated DNA‐PKcs foci colocalized with 53BP1 foci following 10 μm MTX treatment for 60 min. The images in the lower panel are representative of cells in which S2056‐phosphorylated DNA‐PKcs accumulated at discrete nuclear areas that had an absence of 53BP1 signals following 10 μm MTX treatment for 60 min. Typical p‐DNA‐PKcs (S2056) foci outside of DSBs are indicated by arrows. Scale bar, 5 μm. (B) The phosphorylation status of DNA‐PKcs at S2056 was monitored by western blot. (C) A549 cells were transduced with control (sh*NTC*) or sh*DNA‐PKcs* virion for 48 h followed by a 4 h 0.5% DMSO or 10 μm MTX treatment. Protein levels of DNA‐PKcs, S2056‐phosphorylated DNA‐PKcs and GAPDH were monitored by western blot as indicated. (D) Cell lysates from MTX‐treated A549 cells were incubated with calf intestinal alkaline phosphatases (CIAP) for different times. The phosphorylation status of DNA‐PKcs at S2056 was monitored by western blot. (E) A549 cells were transduced with control (sh*NTC*) or sh*DNA‐PKcs* virion for 48 h followed by 10 μm MTX treatment for the indicated times. Representative images of indirect immunostaining of p‐DNA‐PKcs (S2056) are presented. Scale bar, 25 μm.

Mitoxantrone, a synthetic anthracenedione derivative, is well‐characterized as a DNA topoisomerase II (Top II) poison and DNA‐intercalating agent and is commonly applied in the treatment of various cancers 14. To further understand its chemotherapeutic activities, we initially aimed to investigate the MTX‐induced DDR pathway(s) in the human non‐small cell lung cancer cell line A549. As expected, MTX treatment markedly induced genotoxic stress in A549 cells, reflected in a striking increase in the number of cells with nuclear foci accumulating 53BP1, which is a DNA damage repair factor that specifically marks DNA lesions with DSBs (Fig. 1A). Consistently, using a commercial phospho‐specific antibody, we observed a marked induction of phosphorylation on S2056 of DNA‐PKcs following MTX treatment (Fig. 1A,B). It has been firmly established that DNA‐PKcs is specifically autophosphorylated at S2056 after it is recruited to DSBs 10, 11. In agreement with this concept, a substantial fraction of cells contained S2056‐phosphorylated DNA‐PKcs foci colocalized with 53BP1 foci following 10 μm MTX treatment for 60 min (Fig. 1A, upper panel). However, the fluorescence immunostaining assay also showed that S2056‐phosphorylated DNA‐PKcs accumulated at discrete nuclear areas that had an absence of 53BP1 signals in some MTX‐treated A549 cells (Fig. 1A, lower panel).

The specificity of the antibody was confirmed because both the expression of short hairpin RNA (shRNA) targeting *DNA‐PKcs* and phosphatase treatment of cell lysates largely eliminated the signals detected by phospho‐specific DNA‐PKcs antibody in MTX‐treated A549 cells (Fig. 1C,D). Consistently, the fluorescence intensity of S2056‐phosphorylated DNA‐PKcs in the indirect immunostaining assay was severely reduced in the *DNA‐PKcs* shRNA (sh*DNA‐PKcs*) virion‐transduced cells treated with MTX for different times (Fig. 1E).

### MTX induces a dynamic change in nuclear localization of S2056‐phosphorylated DNA‐PKcs

Although the recruitment and stimulation of DNA‐PK at DSBs are essential for the NHEJ initiation, the sustained presence of DNA‐PKcs at DSB lesions interferes with the subsequent DNA‐end ligation. Evidence from both *in vitro* and *in vivo* studies supports that autophosphorylation on S2056 destabilizes the association of DNA‐PKcs with broken DNA ends 8, 11. This finding raises the possibility that S2056‐phosphorylated DNA‐PKcs may be redistributed as it is excluded from the DNA damage sites in MTX‐treated A549 cells.

**Figure 2:**
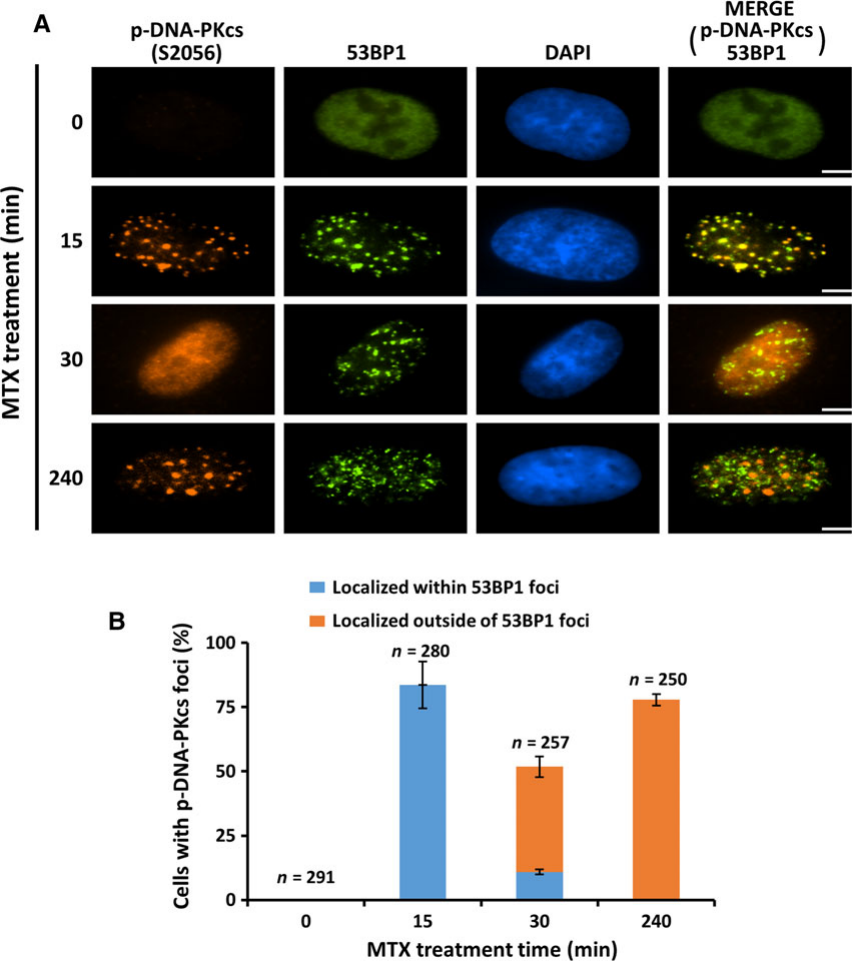
S2056‐phosphorylated DNA‐PKcs accumulates at nuclear areas that lack 53BP1 in MTX‐treated A549 cells. (A) Colocalization of phosphorylated DNA‐PKcs (p‐DNA‐PKcs (S2056)) with nuclear foci localizing 53BP1 was analyzed by indirect immunostaining in A549 cells treated with 10 μm MTX for the indicated times. Representative images are presented. Scale bar, 5 μm. (B) Quantitative analyses of cells with > 90% p‐DNA‐PKcs (S2056) foci localized within or outside of 53BP1 foci. Only cells that carried more than five discrete p‐DNA‐PKcs (S2056) nuclear foci were included in the quantitative analyses. The number of cells scored for each condition is indicated in the graphs. Error bars represent standard deviations from three independent experiments.

**Figure 3:**
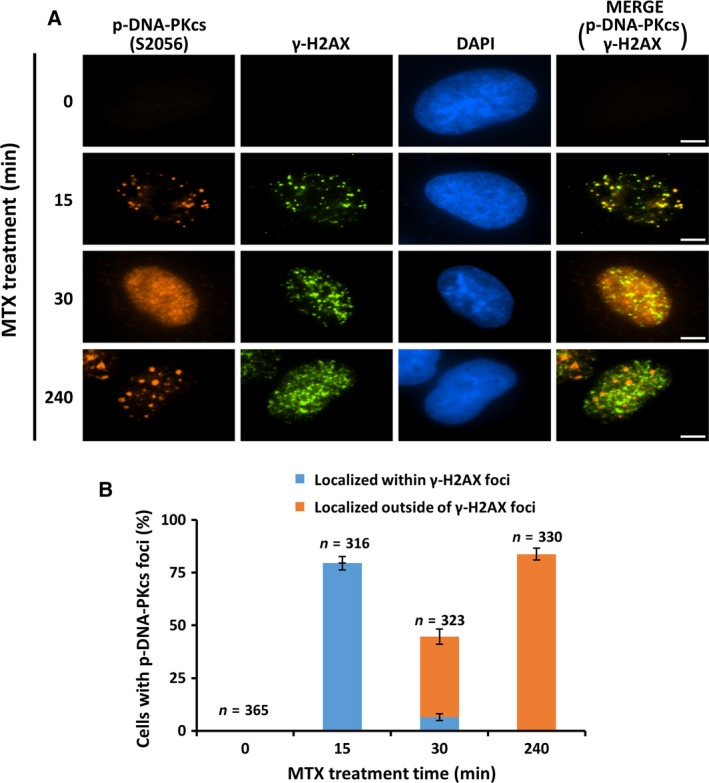
S2056‐phosphorylated DNA‐PKcs (p‐DNA‐PKcs) accumulates at nuclear areas that lack γ‐H2AX in MTX‐treated A549 cells. (A) Colocalization of p‐DNA‐PKcs (S2056) with nuclear foci localizing γ‐H2AX was analyzed by indirect immunostaining in A549 cells treated with 10 μm MTX for the indicated times. Representative images are presented. Scale bar, 5 μm. (B) Quantitative analyses of cells with > 90% p‐DNA‐PKcs (S2056) foci localized within or outside of γ‐H2AX foci. Only cells that carried more than five discrete p‐DNA‐PKcs (S2056) nuclear foci were included in the quantitative analyses. The number of cells scored for each condition is indicated in the graphs. Error bars represent standard deviations from three independent experiments.

**Figure 4:**
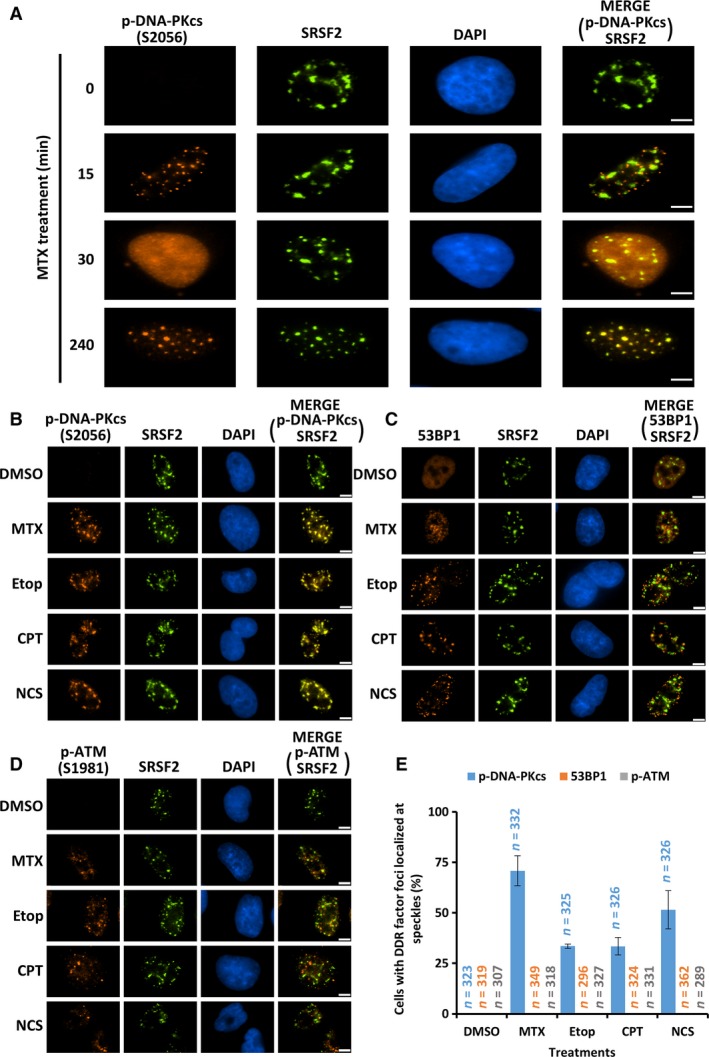
S2056‐phosphorylated DNA‐PKcs is redistributed to nuclear speckles at the late stage of DDR. (A) Colocalization of phosphorylated DNA‐PKcs (p‐DNA‐PKcs (S2056)) with nuclear speckles (SRSF2) was analyzed via indirect immunostaining in A549 cells treated with 10 μm MTX for the indicated times. Representative images are presented. (B–D) Colocalization of p‐DNA‐PKcs (S2056) (B), 53BP1 (C), and p‐ATM (S1981) (D) foci with SRSF2 was analyzed by indirect immunostaining in A549 cells treated with DMSO, 10 μm MTX, 34 μm Etop, 2 μm CPT and 625 ng·mL^−1^ NCS for 4 h, respectively. Representative images are presented. Scale bar, 5 μm. (E) Quantitative analyses of cells with > 90% DDR factor foci colocalized with speckles in (B–D). Only cells that carried more than five discrete DDR factor nuclear foci were included in the quantitative analyses. The number of cells scored for each condition is indicated in the graphs. Error bars represent standard deviations from three independent experiments.

To test this hypothesis, we carefully examined the subcellular localization of S2056‐phosphorylated DNA‐PKcs in A549 cells that were treated with 10 μm MTX for various times. At this dose, S2056 phosphorylation was detected as early as 15 min post‐MTX addition (Figs 1B, 2A, 3A and 4A). Consistent with the notion that autophosphorylation of DNA‐PKcs is stimulated by DNA ends at DSBs, fluorescence immunostaining showed that S2056‐phosphorylated DNA‐PKcs predominantly accumulated at 53BP1‐ and γ‐H2AX‐containing nuclear foci following 15 min of MTX treatment (Figs 2 and 3, 15 min time point). As the MTX treatment was extended, the signals of S2056‐phosphorylated DNA‐PKcs in a substantial fraction of cells became diffused (Figs 2 and 3, 30 min time point). This finding is consistent with the previous observations that the phosphorylation of DNA‐PKcs promotes its dissociation from DNA lesions 8, 11. Intriguingly, discrete S2056‐phosphorylated DNA‐PKcs foci started to occur at nuclear areas that lacked 53BP1 and γ‐H2AX signals following a 30 min MTX treatment (data not shown). Moreover, the number of S2056‐phosphorylated DNA‐PKcs foci outside of DNA lesions sharply increased after a 4 h MTX treatment. At this time point, in about three‐quarters of MTX‐treated cells, > 90% of the S2056‐phosphorylated DNA‐PKcs foci occurred at nuclear areas with an absence of 53BP1 and γ‐H2AX signals (Figs 2 and 3, 240 min time point). These data strongly suggested a dynamic change in the nuclear localizations of S2056‐phosphorylated DNA‐PKcs in MTX‐treated A549 cells.

### S2056‐phosphorylated DNA‐PKcs is translocated to nuclear speckles at the late stage of DDR

To understand the nature of the DSB‐free nuclear foci to which S2056‐phosphorylated DNA‐PKcs is translocated in response to MTX‐induced genotoxic stress, we conducted indirect fluorescence immunostaining analyses to costain S2056‐phosphorylated DNA‐PKcs with proteins marking various nuclear subcompartments. This study led to an unexpected finding that the foci of S2056‐phosphorylated DNA‐PKcs at nuclear areas that lacked DNA lesions were colocalized with nuclear speckles, indicated by a perfect colocalization of S2056‐phosphorylated DNA‐PKcs with splicing factor SRSF2 (Fig. 4A, 240 min time point).

Notably, by examining the colocalization of S2056‐phosphorylated DNA‐PKcs with SRSF2 or DDR factors 53BP1 and γ‐H2AX at various time intervals following MTX treatment, we found that the accumulation of S2056‐phosphorylated DNA‐PKcs at nuclear speckles occurred substantially later than its appearance at DSBs following MTX treatment, which suggests that S2056‐phosphorylated DNA‐PKcs is redistributed to nuclear speckles after it is dissociated from DNA damage sites (Figs 2A, 3A and 4A). Moreover, the accumulation of S2056‐phosphorylated DNA‐PKcs at speckles is not restricted to MTX‐treated A549 cells. It was observed in both A549 and U2OS cells in the presence of various DSB inducers, including etoposide (Etop, topoisomerase II poison inhibitor), camptothecin (CPT, topoisomerase I poison inhibitor) and neocarzinostatin (NCS, a type of radiomimetic) (Fig. 4B,E and data not shown). In contrast, both 53BP1 and activated ATM (phosphorylated at S1981), another phosphoinositide 3‐kinase‐related kinase that is recruited and stimulated at DSBs, were absent at nuclear speckles even up to 4 h of treatment with various DSB inducers in both A549 and U2OS cells (Fig. 4C–E and data not shown). Taken together, our data demonstrated that DSB‐induced genotoxic stress results in the accumulation of autophosphorylated DNA‐PKcs at nuclear speckles in human cells after it is dissociated from DNA damage sites.

### DNA‐PKcs is involved in the modulation of pre‐mRNA alternative splicing in response to MTX‐induced genotoxic stress

Speckles are the nuclear structures that function as storage, assembly and modification sites for numerous factors that modulate mRNA metabolism 15. We have shown that genotoxic stress induces the accumulation of DNA‐PKcs, the catalytic subunit of a DSB‐stimulated serine/threonine protein kinase, at nuclear speckles. One potential outcome of this accumulation could be the involvement of DNA‐PKcs in modulating the activity of speckle‐associated splicing factors in response to genotoxic stress. In this regard, it is of note that the activities of numerous splicing factors are strictly controlled by negative feedback loops, and alternative splicing adds an important layer to the homeostatic control of their activities *in vivo*. For example, elevated activity of the splicing activator(s), such as SRSF1, promotes the excision of an alternatively spliced (AS) intron within the 3′‐UTR of its own transcripts in mammalian cells 16, which generates isoforms that harbor a premature termination codon (PTC), destined to be degraded by the nonsense‐mediated mRNA decay pathway 17. In contrast, when splicing silencer(s), such as polypyrimidine tract‐binding protein, is upregulated, its productive alternative splicing is suppressed 18. We thus decided to assess whether DNA‐PKcs is involved in regulating alternative splicing of certain splicing regulator(s) during the cellular response to genotoxic stress.

**Figure 5:**
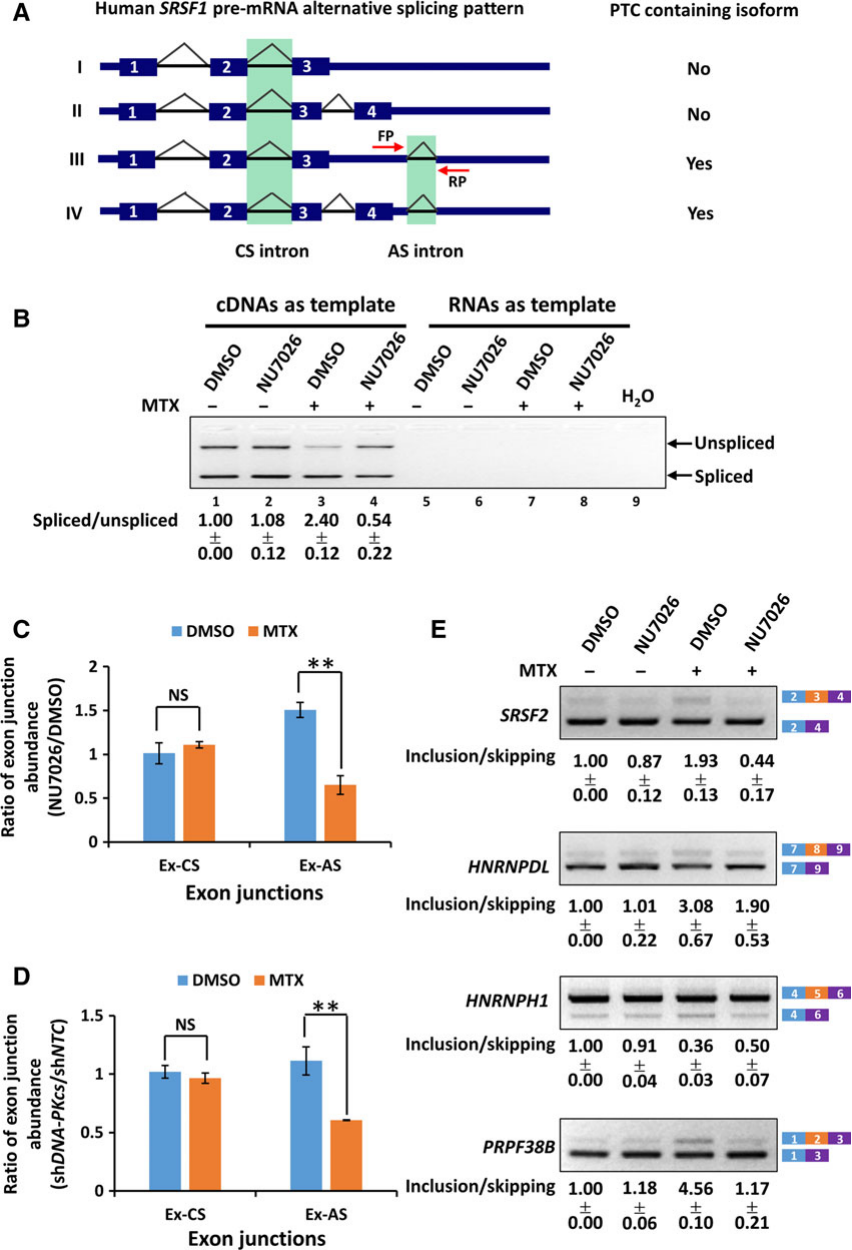
DNA‐PKcs is involved in the control of pre‐mRNA alternative splicing in MTX‐treated A549 cells. (A) Schematic representation of human *SRSF1* pre‐mRNA alternative splicing pattern. Whether the isoform contains a premature termination codon (PTC) was indicated on the right. (B) A549 cells were pretreated with 0.5% DMSO or 20 μm DNA‐PK inhibitor NU7026 for 30 min, followed by a 4 h treatment of DMSO or 10 μm MTX. Regular RT‐PCR was performed to monitor the splicing efficiency of an AS intron of the *SRSF1* pre‐mRNA. Primer locations are indicated in (A) by the red arrows. A representative agarose gel electrophoresis image is presented. The ratio of the signal of the spliced RNA to that of the unspliced isoform is indicated. (C) A549 cells were treated as indicated in (B). qRT‐PCR was applied to monitor the abundance of the indicated spliced exon junctions of the *SRSF1* mRNA using junction specific primers. qRT‐PCR signal of each junction was normalized to that of *SRSF1* exon 1 in parallel assays. Ratios of the normalized junction abundances in the NU7026‐treated cells to that in the DMSO‐treated cells are shown in the graph. (D) A549 cells were transduced with control (sh*NTC*) or sh*DNA‐PKcs* virion for 48 h followed by a 4 h treatment of DMSO or 10 μm MTX. Relative abundance of the indicated exon junction was analyzed by qRT‐PCR as indicated in (C). Error bars represent standard deviations from three independent experiments. Significance of changes in splicing efficiency was assessed using Student's two‐tailed *t* test with significant changes indicated by **P < 0.01; NS, not significant. (E) A549 cells were treated as described in (B). Regular RT‐PCR was performed to monitor splicing of the indicated transcripts. Representative agarose gel electrophoresis images are presented. The ratio of the signal of the RNA carrying the alternative spliced exon (inclusion) to that of the RNA skipping the corresponding exon (skipping) is indicated.

Intriguingly, we found that inactivation of DNA‐PK by both its specific small molecule inhibitor NU7026 and RNA interference specifically decreases the excision of an AS intron localizing at the 3′‐UTR of the human *SRSF1* mRNAs in the MTX‐treated A549 cells (Fig. 5A). As shown in Fig. 5B, NU7026 treatment results in a significant decrease in the ratio of the spliced to the unspliced *SRSF1* mRNA isoforms in the MTX‐treated A549 cells (lane 3 in comparison with lane 4), but not in A549 cells without MTX treatment (lane 1 in comparison with lane 2). Consistently, the abundance of the relative exon junction in the MTX‐treated A549 cells is also decreased in the presence of NU7026 or shRNA specifically targeting DNA‐PKcs (Fig. 5C–D). More importantly, neither DNA‐PK inhibitor nor the expression of the shRNA specifically targeting DNA‐PKcs had an inhibitory effect on the excision of a CS intron of the *SRSF1* transcripts in cells with or without MTX treatment (Fig. 5C–D). Significantly, DNA‐PK inactivation by NU7026 also affects alternative splicing of the *SRSF2*,* HNRNPDL*,* HNRNPH1* and *PRPF38B* transcripts in the MTX‐treated A549 cells (Fig. 5E). Together, these data strongly support an involvement of DNA‐PKcs in controlling pre‐mRNA splicing in response to the MTX‐induced genotoxic stress.

## Discussion

We have demonstrated that a DDR kinase, DNA‐PKcs, can be translocated to nuclear speckles in response to various DSB inducers, which is highly likely after it is stimulated and dissociated from DNA lesions. Indicative of a functional relevance for DNA‐PKcs redistribution to speckles, we further defined a previously uncharacterized role of DNA‐PKcs in modulating pre‐mRNA alternative splicing in the presence of the Top II inhibitor MTX‐induced genotoxic stress. These observations not only shed new light on the complexity of the cellular response to DNA damage, but also indicate a novel mechanism that connects DDR to RNA metabolism in human cells.

Approximately 90% of multiexon human genes undergo alternative splicing, by which a single gene can generate multiple mRNA isoforms with differences in their stability and/or their protein coding information 19. Intriguingly, an increasing body of evidence has demonstrated that alterations in pre‐mRNA splicing play a widespread regulatory role in the functional tuning of transcriptomes in response to DNA damage. For example, genotoxic stress, induced by UV, CPT, doxorubicin and cisplatin, promotes exon(s) skipping in transcripts of the *MDM2* gene, which encodes a p53‐specific E3‐ligase 20, 21, 22. In this case, splicing alterations promote the expression of MDM2 isoforms that lack its interaction domain with p53, which in turn favors p53 activation in response to genotoxic stress. Several recent transcriptome‐wide analyses have indicated an increasing number of alternative splicing events are regulated by various genotoxic inducers, further highlighting the impact of splicing modulation on the cellular response to DNA damage 23, 24, 25. Our finding that DSB‐induced genotoxic stress induces the accumulation of autophosphorylated DNA‐PKcs at nuclear speckles indicates a potential physical connection between DDR and pre‐mRNA splicing during the cellular response to DNA damage. Investigation of the biological relevance of dynamic DNA‐PKcs distribution in response to DSB‐induced genotoxic stress will be an attractive topic for future study.

Of note, our data show that the phosphorylation of DNA‐PKcs at S2056 is persistently maintained even after DNA‐PKcs is dissociated from the damage sites. While DNA ends at DNA damage sites are able to activate the autophosphorylation of DNA‐PKcs at S2056, DNA‐PK can be stimulated by other mechanism(s). Early studies demonstrated that DNA‐PK can be activated and phosphorylate the RNA processing factors heterogeneous nuclear ribonucleoproteins (hnRNPs) in an RNA‐dependent manner 26. In this regard, considering that speckles contain abundant RNA species, the idea that the phosphorylation of DNA‐PK is further stimulated by its associated RNA species after DNA‐PK leaves the damage site is an intriguing possibility to be explored in future studies.

An interesting question raised by our observations is related to how mechanistically DNA‐PK influences pre‐mRNA splicing in the presence of genotoxic stress. Two potential scenarios can be envisioned. In one model, DSB‐activated DNA‐PK may be translocated to speckles and contribute to the control of the phosphorylation status of splicing factors during the cellular response to DNA damage. This idea is consistent with the early observation that DNA‐PK can phosphorylate a number of hnRNPs 26. hnRNPs comprise a family of RNA binding proteins that function in multiple aspects of RNA metabolism, including pre‐mRNA splicing 27. Along these lines, it is interesting to note that both the activity and the cellular distribution of a large number of speckle‐associated splicing factors are regulated by phosphorylation 28. In a second scenario, the accumulation of DNA‐PKcs at speckles may affect the activity of the splicing machinery independent of its kinase activity. This may be analogous to the way that DNA‐PK functions at DNA damage sites in the NHEJ pathway. In this case, although numerous NHEJ factors are putative DNA‐PK substrates, the kinase activity of DNA‐PKcs is only important for its own autophosphorylation and subsequent dissociation from DNA damage sites 8, 10, 11; however, the kinase activity of DNA‐PK is dispensable for the recruitment and activation of downstream factors in the NHEJ pathway 29. It has thus been proposed that DNA‐PKcs most likely provides a physical block to facilitate the formation of a proper juxtaposition of broken ends during the NHEJ process 5. Kinase‐dead DNA‐PKcs mutant cells will be useful to clarify whether a similar mechanism underlies the function of DNA‐PKcs in controlling pre‐mRNA splicing in cellular response to genotoxic stress in future studies.

## Conclusions

In this study, we show that DSB‐induced genotoxic stress triggers a dynamic change in the nuclear localizations of autophosphorylated DNA‐PKcs at S2056 in human cells. Time‐course analyses unexpectedly indicated that S2056‐phosphorylated DNA‐PKcs is translocated to nuclear speckles, structures that consists of numerous factors that modulate mRNA metabolism, highly likely after it has dissociated from DSB lesions. Suggestive of an indispensable role of DNA‐PK in modulating pre‐mRNA splicing in the genotoxic stress response, we found that DNA‐PK inactivation, by both a small molecule inhibitor and RNA interference, affects splicing of a set of pre‐mRNAs in DSB‐inducer MTX‐treated A549 cells. These data thus indicate a novel mechanism that connects DDR to RNA metabolism in human cells.

## Author contributions

XL designed the study. The experiments and data analyses were performed by SL, YS and QW. XL and SL wrote the manuscript, and all authors participated in the discussion and revision of the manuscript.

## Conflict of interest

The authors declare no conflict of interest.
